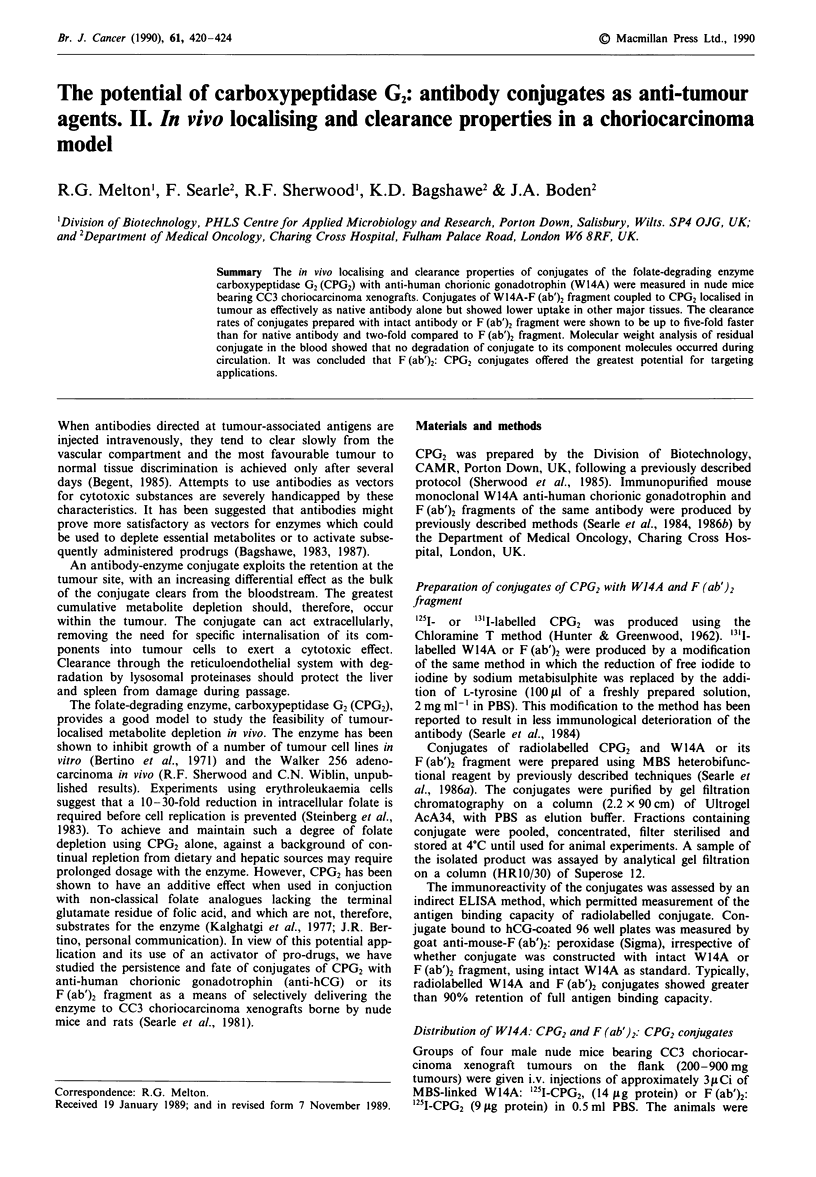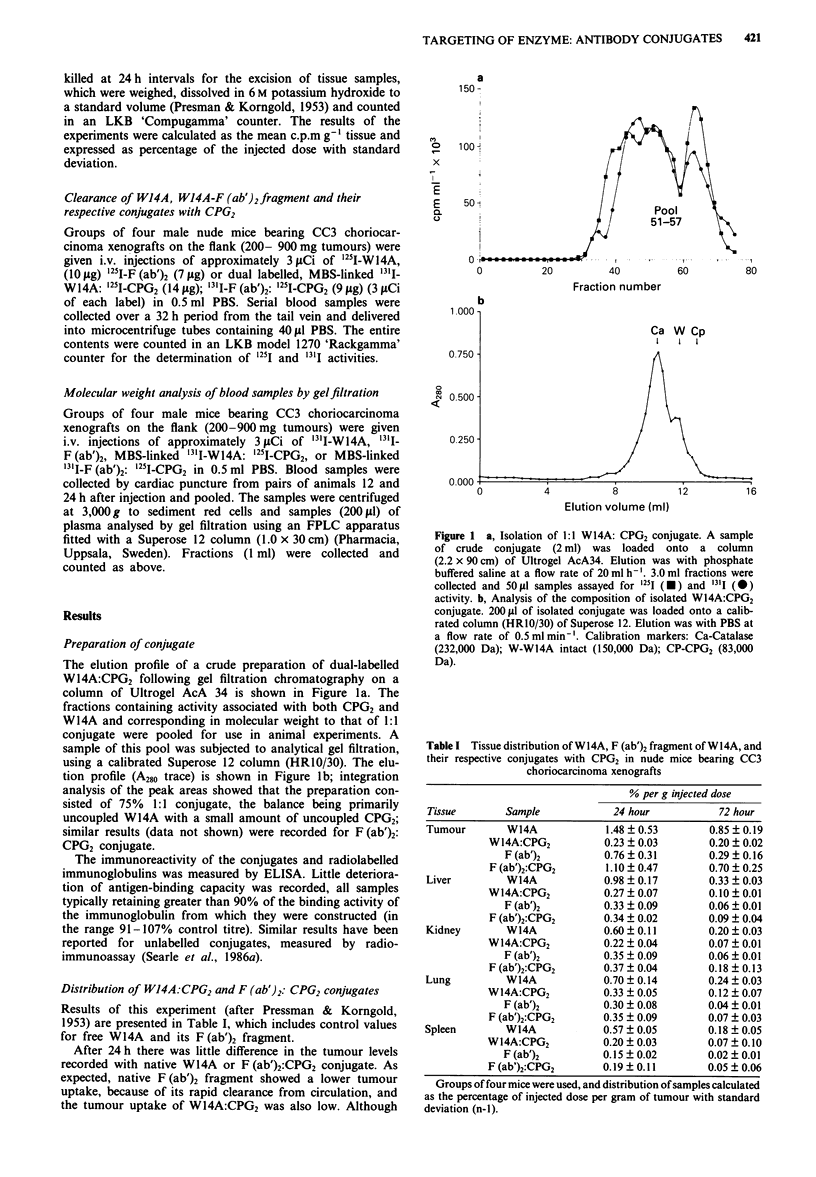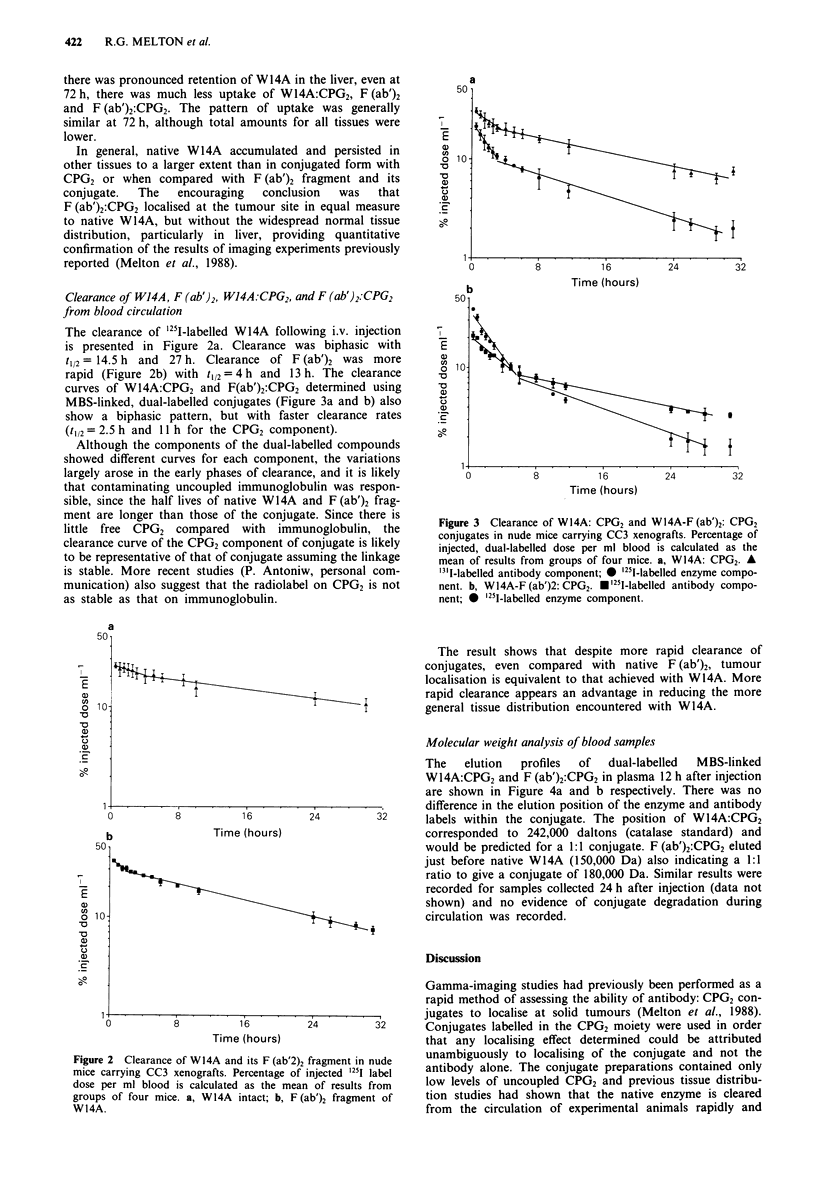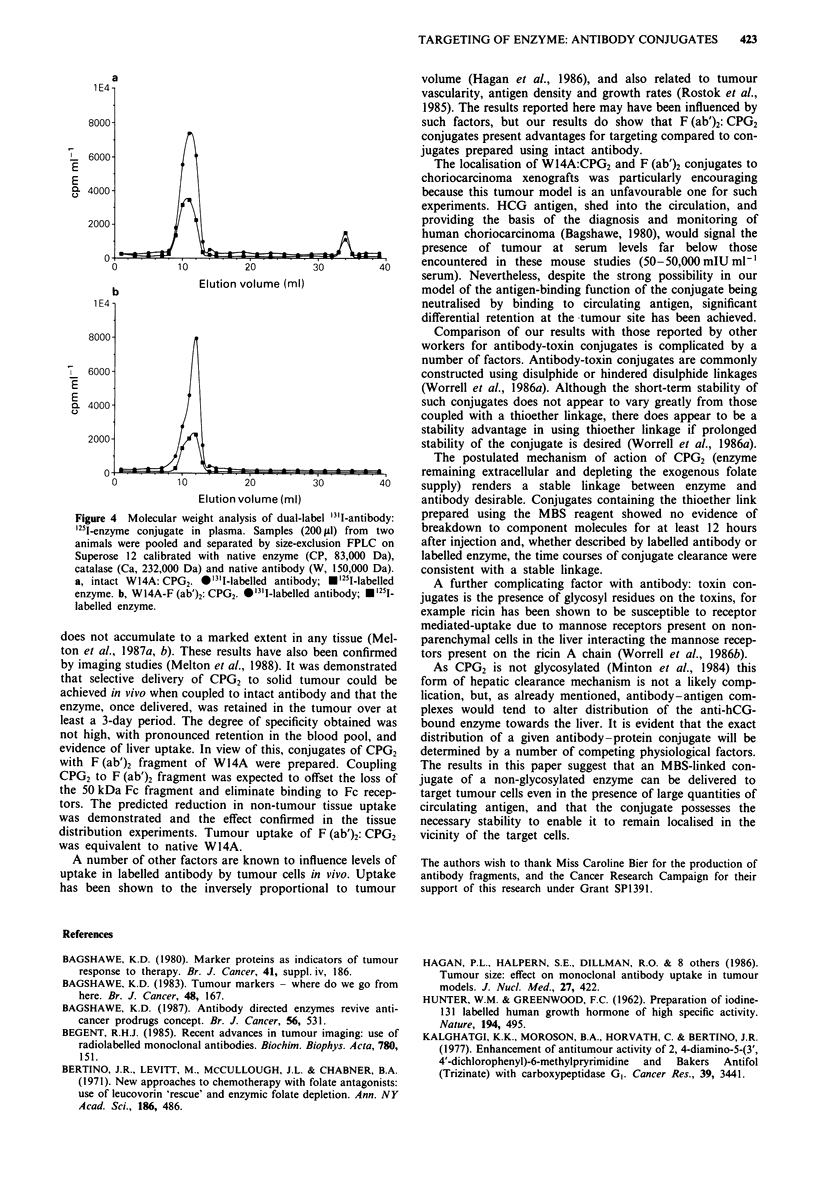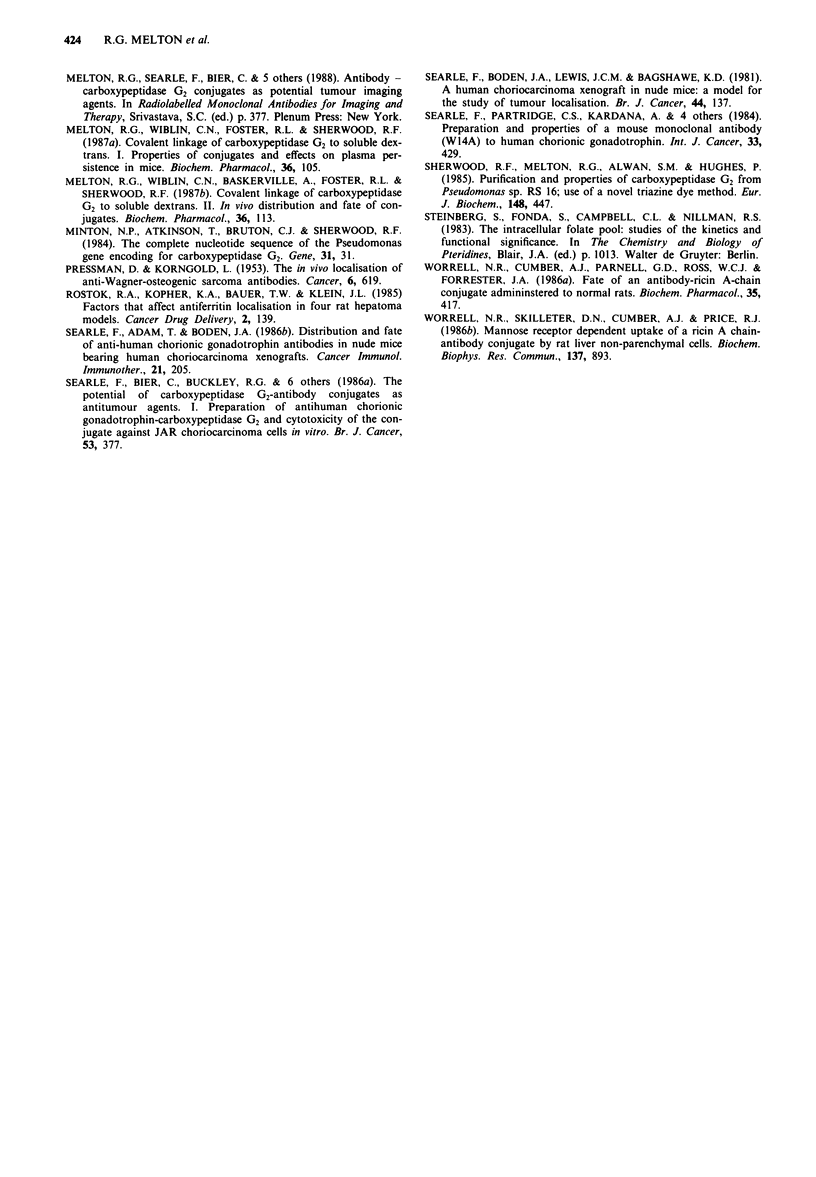# The potential of carboxypeptidase G2: antibody conjugates as anti-tumour agents. II. In vivo localising and clearance properties in a choriocarcinoma model.

**DOI:** 10.1038/bjc.1990.92

**Published:** 1990-03

**Authors:** R. G. Melton, F. Searle, R. F. Sherwood, K. D. Bagshawe, J. A. Boden

**Affiliations:** Division of Biotechnology, PHLS Centre for Applied Microbiology and Research, Salisbury, Wilts, UK.

## Abstract

The in vivo localising and clearance properties of conjugates of the folate-degrading enzyme carboxypeptidase G2 (CPG2) with anti-human chorionic gonadotrophin (W14A) were measured in nude mice bearing CC3 choriocarcinoma xenografts. Conjugates of W14A-F (ab')2 fragment coupled to CPG2 localised in tumour as effectively as native antibody alone but showed lower uptake in other major tissues. The clearance rates of conjugates prepared with intact antibody or F (ab')2 fragment were shown to be up to five-fold faster than for native antibody and two-fold compared to F (ab')2 fragment. Molecular weight analysis of residual conjugate in the blood showed that no degradation of conjugate to its component molecules occurred during circulation. It was concluded that F (ab')2: CPG2 conjugates offered the greatest potential for targeting applications.


Br. J. Cancer (1990), 61, 420-424 © Macmillan Press Ltd., 1990

### The potential of carboxypeptidase $G_2$: antibody conjugates as anti-tumour agents. II. *In vivo* localising and clearance properties in a choriocarcinoma model

R.G. Melton[1], F. Searle[2], R.F. Sherwood[1], K.D. Bagshawe[2] & J.A. Boden[2]

[1]*Division of Biotechnology, PHLS Centre for Applied Microbiology and Research, Porton Down, Salisbury, Wilts. SP4 OJG, UK; and* [2]*Department of Medical Oncology, Charing Cross Hospital, Fulham Palace Road, London W6 8RF, UK.*

**Summary** The *in vivo* localising and clearance properties of conjugates of the folate-degrading enzyme carboxypeptidase $G_2$ ($CPG_2$) with anti-human chorionic gonadotrophin (W14A) were measured in nude mice bearing CC3 choriocarcinoma xenografts. Conjugates of W14A-F $(ab')_2$ fragment coupled to $CPG_2$ localised in tumour as effectively as native antibody alone but showed lower uptake in other major tissues. The clearance rates of conjugates prepared with intact antibody or F $(ab')_2$ fragment were shown to be up to five-fold faster than for native antibody and two-fold compared to F $(ab')_2$ fragment. Molecular weight analysis of residual conjugate in the blood showed that no degradation of conjugate to its component molecules occurred during circulation. It was concluded that F $(ab')_2$: $CPG_2$ conjugates offered the greatest potential for targeting applications.

When antibodies directed at tumour-associated antigens are injected intravenously, they tend to clear slowly from the vascular compartment and the most favourable tumour to normal tissue discrimination is achieved only after several days (Begent, 1985). Attempts to use antibodies as vectors for cytotoxic substances are severely handicapped by these characteristics. It has been suggested that antibodies might prove more satisfactory as vectors for enzymes which could be used to deplete essential metabolites or to activate subsequently administered prodrugs (Bagshawe, 1983, 1987).

An antibody-enzyme conjugate exploits the retention at the tumour site, with an increasing differential effect as the bulk of the conjugate clears from the bloodstream. The greatest cumulative metabolite depletion should, therefore, occur within the tumour. The conjugate can act extracellularly, removing the need for specific internalisation of its components into tumour cells to exert a cytotoxic effect. Clearance through the reticuloendothelial system with degradation by lysosomal proteinases should protect the liver and spleen from damage during passage.

The folate-degrading enzyme, carboxypeptidase $G_2$ ($CPG_2$), provides a good model to study the feasibility of tumour-localised metabolite depletion *in vivo*. The enzyme has been shown to inhibit growth of a number of tumour cell lines *in vitro* (Bertino *et al.*, 1971) and the Walker 256 adenocarcinoma *in vivo* (R.F. Sherwood and C.N. Wiblin, unpublished results). Experiments using erythroleukaemia cells suggest that a 10-30-fold reduction in intracellular folate is required before cell replication is prevented (Steinberg *et al.*, 1983). To achieve and maintain such a degree of folate depletion using $CPG_2$ alone, against a background of continual repletion from dietary and hepatic sources may require prolonged dosage with the enzyme. However, $CPG_2$ has been shown to have an additive effect when used in conjuction with non-classical folate analogues lacking the terminal glutamate residue of folic acid, and which are not, therefore, substrates for the enzyme (Kalghatgi *et al.*, 1977; J.R. Bertino, personal communication). In view of this potential application and its use of an activator of pro-drugs, we have studied the persistence and fate of conjugates of $CPG_2$ with anti-human chorionic gonadotrophin (anti-hCG) or its F $(ab')_2$ fragment as a means of selectively delivering the enzyme to CC3 choriocarcinoma xenografts borne by nude mice and rats (Searle *et al.*, 1981).

Correspondence: R.G. Melton.
Received 19 January 1989; and in revised form 7 November 1989.

### Materials and methods

$CPG_2$ was prepared by the Division of Biotechnology, CAMR, Porton Down, UK, following a previously described protocol (Sherwood *et al.*, 1985). Immunopurified mouse monoclonal W14A anti-human chorionic gonadotrophin and F $(ab')_2$ fragments of the same antibody were produced by previously described methods (Searle *et al.*, 1984, 1986*b*) by the Department of Medical Oncology, Charing Cross Hospital, London, UK.

### *Preparation of conjugates of $CPG_2$ with W14A and F $(ab')_2$ fragment*

$^{125}I$- or $^{131}I$-labelled $CPG_2$ was produced using the Chloramine T method (Hunter & Greenwood, 1962). $^{131}I$-labelled W14A or F $(ab')_2$ were produced by a modification of the same method in which the reduction of free iodide to iodine by sodium metabisulphite was replaced by the addition of L-tyrosine (100 $\mu$l of a freshly prepared solution, 2 mg $ml^{-1}$ in PBS). This modification to the method has been reported to result in less immunological deterioration of the antibody (Searle *et al.*, 1984)

Conjugates of radiolabelled $CPG_2$ and W14A or its F $(ab')_2$ fragment were prepared using MBS heterobifunctional reagent by previously described techniques (Searle *et al.*, 1986*a*). The conjugates were purified by gel filtration chromatography on a column (2.2 × 90 cm) of Ultrogel AcA34, with PBS as elution buffer. Fractions containing conjugate were pooled, concentrated, filter sterilised and stored at 4°C until used for animal experiments. A sample of the isolated product was assayed by analytical gel filtration on a column (HR10/30) of Superose 12.

The immunoreactivity of the conjugates was assessed by an indirect ELISA method, which permitted measurement of the antigen binding capacity of radiolabelled conjugate. Conjugate bound to hCG-coated 96 well plates was measured by goat anti-mouse-F $(ab')_2$: peroxidase (Sigma), irrespective of whether conjugate was constructed with intact W14A or F $(ab')_2$ fragment, using intact W14A as standard. Typically, radiolabelled W14A and F $(ab')_2$ conjugates showed greater than 90% retention of full antigen binding capacity.

### *Distribution of W14A: $CPG_2$ and F $(ab')_2$: $CPG_2$ conjugates*

Groups of four male nude mice bearing CC3 choriocarcinoma xenograft tumours on the flank (200-900 mg tumours) were given i.v. injections of approximately 3 $\mu$Ci of MBS-linked W14A: $^{125}I$-$CPG_2$, (14 $\mu$g protein) or F $(ab')_2$: $^{125}I$-$CPG_2$ (9 $\mu$g protein) in 0.5 ml PBS. The animals were killed at 24 h intervals for the excision of tissue samples, which were weighed, dissolved in 6 M potassium hydroxide to a standard volume (Presman & Korngold, 1953) and counted in an LKB 'Compugamma' counter. The results of the experiments were calculated as the mean c.p.m $g^{-1}$ tissue and expressed as percentage of the injected dose with standard deviation.

### *Clearance of W14A, W14A-F (ab')$_2$ fragment and their respective conjugates with CPG$_2$*

Groups of four male nude mice bearing CC3 choriocarcinoma xenografts on the flank (200– 900 mg tumours) were given i.v. injections of approximately 3 $\mu$Ci of $^{125}$I-W14A, (10 $\mu$g) $^{125}$I-F (ab')$_2$ (7 $\mu$g) or dual labelled, MBS-linked $^{131}$I-W14A: $^{125}$I-CPG$_2$ (14 $\mu$g); $^{131}$I-F (ab')$_2$: $^{125}$I-CPG$_2$ (9 $\mu$g) (3 $\mu$Ci of each label) in 0.5 ml PBS. Serial blood samples were collected over a 32 h period from the tail vein and delivered into microcentrifuge tubes containing 40 $\mu$l PBS. The entire contents were counted in an LKB model 1270 'Rackgamma' counter for the determination of $^{125}$I and $^{131}$I activities.

### *Molecular weight analysis of blood samples by gel filtration*

Groups of four male mice bearing CC3 choriocarcinoma xenografts on the flank (200–900 mg tumours) were given i.v. injections of approximately 3 $\mu$Ci of $^{131}$I-W14A, $^{131}$I-F (ab')$_2$, MBS-linked $^{131}$I-W14A: $^{125}$I-CPG$_2$, or MBS-linked $^{131}$I-F (ab')$_2$: $^{125}$I-CPG$_2$ in 0.5 ml PBS. Blood samples were collected by cardiac puncture from pairs of animals 12 and 24 h after injection and pooled. The samples were centrifuged at 3,000 *g* to sediment red cells and samples (200 $\mu$l) of plasma analysed by gel filtration using an FPLC apparatus fitted with a Superose 12 column (1.0 × 30 cm) (Pharmacia, Uppsala, Sweden). Fractions (1 ml) were collected and counted as above.

### Results

### *Preparation of conjugate*

The elution profile of a crude preparation of dual-labelled W14A:CPG$_2$ following gel filtration chromatography on a column of Ultrogel AcA 34 is shown in Figure 1a. The fractions containing activity associated with both CPG$_2$ and W14A and corresponding in molecular weight to that of 1:1 conjugate were pooled for use in animal experiments. A sample of this pool was subjected to analytical gel filtration, using a calibrated Superose 12 column (HR10/30). The elution profile ($A_{280}$ trace) is shown in Figure 1b; integration analysis of the peak areas showed that the preparation consisted of 75% 1:1 conjugate, the balance being primarily uncoupled W14A with a small amount of uncoupled CPG$_2$; similar results (data not shown) were recorded for F (ab')$_2$: CPG$_2$ conjugate.

The immunoreactivity of the conjugates and radiolabelled immunoglobulins was measured by ELISA. Little deterioration of antigen-binding capacity was recorded, all samples typically retaining greater than 90% of the binding activity of the immunoglobulin from which they were constructed (in the range 91–107% control titre). Similar results have been reported for unlabelled conjugates, measured by radioimmunoassay (Searle *et al.*, 1986*a*).

### *Distribution of W14A:CPG$_2$ and F (ab')$_2$: CPG$_2$ conjugates*

Results of this experiment (after Pressman & Korngold, 1953) are presented in Table I, which includes control values for free W14A and its F (ab')$_2$ fragment.

After 24 h there was little difference in the tumour levels recorded with native W14A or F (ab')$_2$:CPG$_2$ conjugate. As expected, native F (ab')$_2$ fragment showed a lower tumour uptake, because of its rapid clearance from circulation, and the tumour uptake of W14A:CPG$_2$ was also low. Although

**Figure 1** **a**, Isolation of 1:1 W14A: CPG$_2$ conjugate. A sample of crude conjugate (2 ml) was loaded onto a column (2.2 × 90 cm) of Ultrogel AcA34. Elution was with phosphate buffered saline at a flow rate of 20 ml $h^{-1}$. 3.0 ml fractions were collected and 50 $\mu$l samples assayed for $^{125}$I (■) and $^{131}$I (●) activity. **b**, Analysis of the composition of isolated W14A:CPG$_2$ conjugate. 200 $\mu$l of isolated conjugate was loaded onto a calibrated column (HR10/30) of Superose 12. Elution was with PBS at a flow rate of 0.5 ml $min^{-1}$. Calibration markers: Ca-Catalase (232,000 Da); W-W14A intact (150,000 Da); CP-CPG$_2$ (83,000 Da).

**Table I** Tissue distribution of W14A, F (ab')$_2$ fragment of W14A, and their respective conjugates with CPG$_2$ in nude mice bearing CC3 choriocarcinoma xenografts

| *Tissue* | *Sample* | *% per g injected dose* 24 hour | 72 hour |
|---|---|---|---|
| Tumour | W14A | 1.48 ± 0.53 | 0.85 ± 0.19 |
| | W14A:CPG$_2$ | 0.23 ± 0.03 | 0.20 ± 0.02 |
| | F (ab')$_2$ | 0.76 ± 0.31 | 0.29 ± 0.16 |
| | F (ab')$_2$:CPG$_2$ | 1.10 ± 0.47 | 0.70 ± 0.25 |
| Liver | W14A | 0.98 ± 0.17 | 0.33 ± 0.03 |
| | W14A:CPG$_2$ | 0.27 ± 0.07 | 0.10 ± 0.01 |
| | F (ab')$_2$ | 0.33 ± 0.09 | 0.06 ± 0.01 |
| | F (ab')$_2$:CPG$_2$ | 0.34 ± 0.02 | 0.09 ± 0.04 |
| Kidney | W14A | 0.60 ± 0.11 | 0.20 ± 0.03 |
| | W14A:CPG$_2$ | 0.22 ± 0.04 | 0.07 ± 0.01 |
| | F (ab')$_2$ | 0.35 ± 0.09 | 0.06 ± 0.01 |
| | F (ab')$_2$:CPG$_2$ | 0.37 ± 0.04 | 0.18 ± 0.13 |
| Lung | W14A | 0.70 ± 0.14 | 0.24 ± 0.03 |
| | W14A:CPG$_2$ | 0.33 ± 0.05 | 0.12 ± 0.07 |
| | F (ab')$_2$ | 0.30 ± 0.08 | 0.04 ± 0.01 |
| | F (ab')$_2$:CPG$_2$ | 0.35 ± 0.09 | 0.07 ± 0.03 |
| Spleen | W14A | 0.57 ± 0.05 | 0.18 ± 0.05 |
| | W14A:CPG$_2$ | 0.20 ± 0.03 | 0.07 ± 0.10 |
| | F (ab')$_2$ | 0.15 ± 0.02 | 0.02 ± 0.01 |
| | F (ab')$_2$:CPG$_2$ | 0.19 ± 0.11 | 0.05 ± 0.06 |

Groups of four mice were used, and distribution of samples calculated as the percentage of injected dose per gram of tumour with standard deviation (n-1).

there was pronounced retention of W14A in the liver, even at 72 h, there was much less uptake of W14A:$CPG_2$, F(ab')$_2$ and F(ab')$_2$:$CPG_2$. The pattern of uptake was generally similar at 72 h, although total amounts for all tissues were lower.

In general, native W14A accumulated and persisted in other tissues to a larger extent than in conjugated form with $CPG_2$ or when compared with F(ab')$_2$ fragment and its conjugate. The encouraging conclusion was that F(ab')$_2$:$CPG_2$ localised at the tumour site in equal measure to native W14A, but without the widespread normal tissue distribution, particularly in liver, providing quantitative confirmation of the results of imaging experiments previously reported (Melton *et al.*, 1988).

### *Clearance of W14A, F(ab')$_2$, W14A:$CPG_2$, and F(ab')$_2$:$CPG_2$ from blood circulation*

The clearance of $^{125}$I-labelled W14A following i.v. injection is presented in Figure 2a. Clearance was biphasic with $t_{1/2}$ = 14.5 h and 27 h. Clearance of F(ab')$_2$ was more rapid (Figure 2b) with $t_{1/2}$ = 4 h and 13 h. The clearance curves of W14A:$CPG_2$ and F(ab')$_2$:$CPG_2$ determined using MBS-linked, dual-labelled conjugates (Figure 3a and b) also show a biphasic pattern, but with faster clearance rates ($t_{1/2}$ = 2.5 h and 11 h for the $CPG_2$ component).

Although the components of the dual-labelled compounds showed different curves for each component, the variations largely arose in the early phases of clearance, and it is likely that contaminating uncoupled immunoglobulin was responsible, since the half lives of native W14A and F(ab')$_2$ fragment are longer than those of the conjugate. Since there is little free $CPG_2$ compared with immunoglobulin, the clearance curve of the $CPG_2$ component of conjugate is likely to be representative of that of conjugate assuming the linkage is stable. More recent studies (P. Antoniw, personal communication) also suggest that the radiolabel on $CPG_2$ is not as stable as that on immunoglobulin.

**Figure 3** Clearance of W14A: $CPG_2$ and W14A-F(ab')$_2$: $CPG_2$ conjugates in nude mice carrying CC3 xenografts. Percentage of injected, dual-labelled dose per ml blood is calculated as the mean of results from groups of four mice. **a**, W14A: $CPG_2$. ▲ $^{131}$I-labelled antibody component; ● $^{125}$I-labelled enzyme component. **b**, W14A-F(ab')2: $CPG_2$. ■ $^{125}$I-labelled antibody component; ● $^{125}$I-labelled enzyme component.

**Figure 2** Clearance of W14A and its F(ab'2)$_2$ fragment in nude mice carrying CC3 xenografts. Percentage of injected $^{125}$I label dose per ml blood is calculated as the mean of results from groups of four mice. **a**, W14A intact; **b**, F(ab')$_2$ fragment of W14A.

The result shows that despite more rapid clearance of conjugates, even compared with native F(ab')$_2$, tumour localisation is equivalent to that achieved with W14A. More rapid clearance appears an advantage in reducing the more general tissue distribution encountered with W14A.

### *Molecular weight analysis of blood samples*

The elution profiles of dual-labelled MBS-linked W14A:$CPG_2$ and F(ab')$_2$:$CPG_2$ in plasma 12 h after injection are shown in Figure 4a and b respectively. There was no difference in the elution position of the enzyme and antibody labels within the conjugate. The position of W14A:$CPG_2$ corresponded to 242,000 daltons (catalase standard) and would be predicted for a 1:1 conjugate. F(ab')$_2$:$CPG_2$ eluted just before native W14A (150,000 Da) also indicating a 1:1 ratio to give a conjugate of 180,000 Da. Similar results were recorded for samples collected 24 h after injection (data not shown) and no evidence of conjugate degradation during circulation was recorded.

### Discussion

Gamma-imaging studies had previously been performed as a rapid method of assessing the ability of antibody: $CPG_2$ conjugates to localise at solid tumours (Melton *et al.*, 1988). Conjugates labelled in the $CPG_2$ moiety were used in order that any localising effect determined could be attributed unambiguously to localising of the conjugate and not the antibody alone. The conjugate preparations contained only low levels of uncoupled $CPG_2$ and previous tissue distribution studies had shown that the native enzyme is cleared from the circulation of experimental animals rapidly and

**Figure 4** Molecular weight analysis of dual-label $^{131}$I-antibody: $^{125}$I-enzyme conjugate in plasma. Samples (200 μl) from two animals were pooled and separated by size-exclusion FPLC on Superose 12 calibrated with native enzyme (CP, 83,000 Da), catalase (Ca, 232,000 Da) and native antibody (W, 150,000 Da). **a**, intact W14A: $CPG_2$. ● $^{131}$I-labelled antibody; ■ $^{125}$I-labelled enzyme. **b**, W14A-F (ab')$_2$: $CPG_2$. ● $^{131}$I-labelled antibody; ■ $^{125}$I-labelled enzyme.

does not accumulate to a marked extent in any tissue (Melton *et al.*, 1987*a*, *b*). These results have also been confirmed by imaging studies (Melton *et al.*, 1988). It was demonstrated that selective delivery of $CPG_2$ to solid tumour could be achieved *in vivo* when coupled to intact antibody and that the enzyme, once delivered, was retained in the tumour over at least a 3-day period. The degree of specificity obtained was not high, with pronounced retention in the blood pool, and evidence of liver uptake. In view of this, conjugates of $CPG_2$ with F (ab')$_2$ fragment of W14A were prepared. Coupling $CPG_2$ to F (ab')$_2$ fragment was expected to offset the loss of the 50 kDa Fc fragment and eliminate binding to Fc receptors. The predicted reduction in non-tumour tissue uptake was demonstrated and the effect confirmed in the tissue distribution experiments. Tumour uptake of F (ab')$_2$: $CPG_2$ was equivalent to native W14A.

A number of other factors are known to influence levels of uptake in labelled antibody by tumour cells *in vivo*. Uptake has been shown to the inversely proportional to tumour volume (Hagan *et al.*, 1986), and also related to tumour vascularity, antigen density and growth rates (Rostok *et al.*, 1985). The results reported here may have been influenced by such factors, but our results do show that F (ab')$_2$: $CPG_2$ conjugates present advantages for targeting compared to conjugates prepared using intact antibody.

The localisation of W14A:$CPG_2$ and F (ab')$_2$ conjugates to choriocarcinoma xenografts was particularly encouraging because this tumour model is an unfavourable one for such experiments. HCG antigen, shed into the circulation, and providing the basis of the diagnosis and monitoring of human choriocarcinoma (Bagshawe, 1980), would signal the presence of tumour at serum levels far below those encountered in these mouse studies (50–50,000 mIU ml$^{-1}$ serum). Nevertheless, despite the strong possibility in our model of the antigen-binding function of the conjugate being neutralised by binding to circulating antigen, significant differential retention at the tumour site has been achieved.

Comparison of our results with those reported by other workers for antibody-toxin conjugates is complicated by a number of factors. Antibody-toxin conjugates are commonly constructed using disulphide or hindered disulphide linkages (Worrell *et al.*, 1986*a*). Although the short-term stability of such conjugates does not appear to vary greatly from those coupled with a thioether linkage, there does appear to be a stability advantage in using thioether linkage if prolonged stability of the conjugate is desired (Worrell *et al.*, 1986*a*).

The postulated mechanism of action of $CPG_2$ (enzyme remaining extracellular and depleting the exogenous folate supply) renders a stable linkage between enzyme and antibody desirable. Conjugates containing the thioether link prepared using the MBS reagent showed no evidence of breakdown to component molecules for at least 12 hours after injection and, whether described by labelled antibody or labelled enzyme, the time courses of conjugate clearance were consistent with a stable linkage.

A further complicating factor with antibody: toxin conjugates is the presence of glycosyl residues on the toxins, for example ricin has been shown to be susceptible to receptor mediated-uptake due to mannose receptors present on non-parenchymal cells in the liver interacting the mannose receptors present on the ricin A chain (Worrell *et al.*, 1986*b*).

As $CPG_2$ is not glycosylated (Minton *et al.*, 1984) this form of hepatic clearance mechanism is not a likely complication, but, as already mentioned, antibody–antigen complexes would tend to alter distribution of the anti-hCG-bound enzyme towards the liver. It is evident that the exact distribution of a given antibody–protein conjugate will be determined by a number of competing physiological factors. The results in this paper suggest that an MBS-linked conjugate of a non-glycosylated enzyme can be delivered to target tumour cells even in the presence of large quantities of circulating antigen, and that the conjugate possesses the necessary stability to enable it to remain localised in the vicinity of the target cells.

The authors wish to thank Miss Caroline Bier for the production of antibody fragments, and the Cancer Research Campaign for their support of this research under Grant SP1391.

### References

BAGSHAWE, K.D. (1980). Marker proteins as indicators of tumour response to therapy. *Br. J. Cancer*, **41**, suppl. iv, 186.

BAGSHAWE, K.D. (1983). Tumour markers – where do we go from here. *Br. J. Cancer*, **48**, 167.

BAGSHAWE, K.D. (1987). Antibody directed enzymes revive anti-cancer prodrugs concept. *Br. J. Cancer*, **56**, 531.

BEGENT, R.H.J. (1985). Recent advances in tumour imaging: use of radiolabelled monoclonal antibodies. *Biochim. Biophys. Acta*, **780**, 151.

BERTINO, J.R., LEVITT, M., McCULLOUGH, J.L. & CHABNER, B.A. (1971). New approaches to chemotherapy with folate antagonists: use of leucovorin 'rescue' and enzymic folate depletion. *Ann. NY Acad. Sci.*, **186**, 486.

HAGAN, P.L., HALPERN, S.E., DILLMAN, R.O. & 8 others (1986). Tumour size: effect on monoclonal antibody uptake in tumour models. *J. Nucl. Med.*, **27**, 422.

HUNTER, W.M. & GREENWOOD, F.C. (1962). Preparation of iodine-131 labelled human growth hormone of high specific activity. *Nature*, **194**, 495.

KALGHATGI, K.K., MOROSON, B.A., HORVATH, C. & BERTINO, J.R. (1977). Enhancement of antitumour activity of 2, 4-diamino-5-(3', 4'-dichlorophenyl)-6-methylpryrimidine and Bakers Antifol (Trizinate) with carboxypeptidase $G_1$. *Cancer Res.*, **39**, 3441.

MELTON, R.G., SEARLE, F., BIER, C. & 5 others (1988). Antibody – carboxypeptidase $G_2$ conjugates as potential tumour imaging agents. In *Radiolabelled Monoclonal Antibodies for Imaging and Therapy*, Srivastava, S.C. (ed.) p. 377. Plenum Press: New York.

MELTON, R.G., WIBLIN, C.N., FOSTER, R.L. & SHERWOOD, R.F. (1987*a*). Covalent linkage of carboxypeptidase $G_2$ to soluble dextrans. I. Properties of conjugates and effects on plasma persistence in mice. *Biochem. Pharmacol.*, **36**, 105.

MELTON, R.G., WIBLIN, C.N., BASKERVILLE, A., FOSTER, R.L. & SHERWOOD, R.F. (1987*b*). Covalent linkage of carboxypeptidase $G_2$ to soluble dextrans. II. *In vivo* distribution and fate of conjugates. *Biochem. Pharmacol.*, **36**, 113.

MINTON, N.P., ATKINSON, T., BRUTON, C.J. & SHERWOOD, R.F. (1984). The complete nucleotide sequence of the Pseudomonas gene encoding for carboxypeptidase $G_2$. *Gene*, **31**, 31.

PRESSMAN, D. & KORNGOLD, L. (1953). The *in vivo* localisation of anti-Wagner-osteogenic sarcoma antibodies. *Cancer*, **6**, 619.

ROSTOK, R.A., KOPHER, K.A., BAUER, T.W. & KLEIN, J.L. (1985) Factors that affect antiferritin localisation in four rat hepatoma models. *Cancer Drug Delivery*, **2**, 139.

SEARLE, F., ADAM, T. & BODEN, J.A. (1986*b*). Distribution and fate of anti-human chorionic gonadotrophin antibodies in nude mice bearing human choriocarcinoma xenografts. *Cancer Immunol. Immunother.*, **21**, 205.

SEARLE, F., BIER, C., BUCKLEY, R.G. & 6 others (1986*a*). The potential of carboxypeptidase $G_2$-antibody conjugates as antitumour agents. I. Preparation of antihuman chorionic gonadotrophin-carboxypeptidase $G_2$ and cytotoxicity of the conjugate against JAR choriocarcinoma cells *in vitro*. *Br. J. Cancer*, **53**, 377.

SEARLE, F., BODEN, J.A., LEWIS, J.C.M. & BAGSHAWE, K.D. (1981). A human choriocarcinoma xenograft in nude mice: a model for the study of tumour localisation. *Br. J. Cancer*, **44**, 137.

SEARLE, F., PARTRIDGE, C.S., KARDANA, A. & 4 others (1984). Preparation and properties of a mouse monoclonal antibody (W14A) to human chorionic gonadotrophin. *Int. J. Cancer*, **33**, 429.

SHERWOOD, R.F., MELTON, R.G., ALWAN, S.M. & HUGHES, P. (1985). Purification and properties of carboxypeptidase $G_2$ from *Pseudomonas* sp. RS 16; use of a novel triazine dye method. *Eur. J. Biochem.*, **148**, 447.

STEINBERG, S., FONDA, S., CAMPBELL, C.L. & NILLMAN, R.S. (1983). The intracellular folate pool: studies of the kinetics and functional significance. In *The Chemistry and Biology of Pteridines*, Blair, J.A. (ed.) p. 1013. Walter de Gruyter: Berlin.

WORRELL, N.R., CUMBER, A.J., PARNELL, G.D., ROSS, W.C.J. & FORRESTER, J.A. (1986*a*). Fate of an antibody-ricin A-chain conjugate admininstered to normal rats. *Biochem. Pharmacol.*, **35**, 417.

WORRELL, N.R., SKILLETER, D.N., CUMBER, A.J. & PRICE, R.J. (1986*b*). Mannose receptor dependent uptake of a ricin A chain-antibody conjugate by rat liver non-parenchymal cells. *Biochem. Biophys. Res. Commun.*, **137**, 893.